# Bibliometric dataset (1995–2022) on green jobs: A comprehensive analysis of scientific publications

**DOI:** 10.1016/j.dib.2023.109845

**Published:** 2023-12-07

**Authors:** Alexandre Mathieu

**Affiliations:** UMI SOURCE, Soutenabilité et Résilience (Université Paris-Saclay, UVSQ, IRD), 78280, Guyancourt, France

**Keywords:** Bibliometrics, Green Job, Green collar job, Green work, Green employment, Green economy, Sustainable development

## Abstract

The realm of green jobs presents a fertile ground for understanding the intersecting pathways between sustainable transition and the labor market. We have crafted a bibliometric dataset centered on this concept, amassing 414 articles from the Scopus and Web of Science databases, following a laid down protocol, PRISMA, spanning the period from 1995 to 2022. This endeavor aims to depict the dynamics, themes, and conceptual approaches shaping the discourse on green jobs. The dataset, structured around 13 descriptive variables such as authors, keywords, and cited references, is made available to researchers, institutions, and decision-makers to provide insight into the academic debates on ecological transition through the lens of employment, especially in the wake of a green economy. The potential for reutilizing these data is expansive. They can serve as a foundation for comparative analyses with the media and institutional portrayals of green jobs. Furthermore, the dataset can be enriched by integrating other forms of literature, such as books, chapters, or conference proceedings, while retaining the existing structure. This expansibility paves the way for a multidisciplinary and multilingual exploration, thereby enhancing the richness and diversity of possible analyses.

Specifications Table

**Table:** 

Subject	Economic Development and Growth
Specific subject area	Bibliometric metadata sourced from Scopus and Web of Science databases, pertaining to academic literature articles focused exclusively on green jobs.
Data format	**Raw, Analyzed, Filtered** (in .CSV)
Type of data	**Table** (in .CSV)
Data collection	Raw data was compiled by merging results from similar searches on the Scopus and Web of Science databases. The merging process and removal of duplicates were facilitated using the Bibliometrix software and Excel. No time constraints were applied, and initially, the maximum number of variables was retained. Variable selection was later based on their presumed relevance. Article sorting was conducted following the PRISMA protocol, involving a review of abstracts and, when necessary, the full articles. The entire database is consolidated into a single spreadsheet.
Data source location	The collected data originate from the electronic repositories Scopus and Web of Science, focusing on research articles written in English, with no temporal period restrictions. Institution: Université Versailles Saint-Quentin-en-Yvelines (UVSQ) – Université Paris-Saclay Address: 47 boulevard Vauban, 78280, Guyancourt Cedex Country: France Electronic database: Scopus (https://www-scopus-com.ezproxy.universite-paris-saclay.fr/search/form.uri?display=basic#basic) and Web of Science (https://www-webofscience-com.ezproxy.universite-paris-saclay.fr)
Data accessibility	Repository name: RechercheDataGouv Saclay repository Data identification number: https://doi.org/10.57745/4LOIZB Direct URL to data: https://entrepot.recherche.data.gouv.fr/dataset.xhtml?persistentId=doi:10.57745/4LOIZB

## 1 Value of the Data

• These data are valuable as they provide an in-depth analysis of publications in scientific journals on green jobs, revealing trends, themes, conceptual pillars, dans outlooks in this field. They shed light on discussions about ecological transition and employment, especially within the context of a green economy. Delving into these data can also highlight gaps in current research and steer the course of future academic studies.
• Various stakeholders stand to gain. Researchers, academics, institutions, and decision-makers can harness it to deepen their understanding of green job-related issues without the need for preliminary sorting to obtain pertinent texts.
• Rooted in a comprehensive analysis of international political economy, the data underscore the evolution of the green job concept, offering academic insights. A comparison with media and institutional portrayals of this idea would be relevant. highlighting the evolution of the concept of green jobs. They provide an academic insight into this topic. Comparing this foundation with the media and institutional representations of this concept would be apt.
• Comprising articles from scientific journals, the dataset can be expanded with books, chapters, or conference proceedings, while maintaining the same structure. Contributions in other languages or from journals not indexed on Scopus and Web of Science can also be included.

## 2 Background

This bibliometric data set has been developed as part of a study on the conceptual foundations of green jobs and their academic representation. It aims to explore the resonance and compatibility of this concept in various contexts, such as those of the Global North and the Global South. To date, the concept has suffered from a lack of a consensual definition of green jobs, presenting a significant challenge and making the concept difficult to operationalize. However, the definition provided by the ILO and the United Nations in 2008 is often used as a benchmark, identifying green jobs as key positions across numerous sectors that are essential for the preservation and conservation of the environment, while also promoting the creation of decent jobs [1]. In the face of current environmental, economic, and social challenges, green jobs fluctuate in the literature between being agents of short-term economic transformation and elements of a sustainable and inclusive green economy. This ambivalence highlights the need for a comprehensive approach to their integration into sustainable development strategies. This project is part of a broader effort to assess the role of green jobs in sustainable development, their potential to reconcile environmental preservation, economic advancement, and social justice.

## 3 Data Description

The database, named “BDGJ” as an acronym for Bibliometric Dataset on Green Jobs [2], primarily consists of a dataset titled “BDGJ_dataset.csv”. This file includes a single sheet, also named “BDGJ_dataset”, which compiles information pertaining to 414 articles written in English, exclusively derived from the scientific databases Scopus and Web of Science, spanning from the year 1995 to 2022. The dataset encompasses 13 descriptive variables, titled in accordance with the field tags from the Web of Science Core Collection, represented as two-character tags. Table 1 provides a description of these variables within the data corpus and details their significance and the analytical possibilities each variable offers. Additionally, a separate file, named “BDGJ_variables.txt”, outlines the definitions of each variable.

**Table 1:** Description of the 13 variables of BDGJ.

Two-character field tags	Signification	Description	Variable Interest
AU	Authors	Enter the last name first followed by a space and the author's initials.	Analysis of co-authorship networks, identification of prolific authors, and institutional and international collaboration.
DE	Author keywords	Refers to the keywords provided directly by the authors of the articles.	Trend analysis of research topics, identification of emerging themes, and mapping of research fields.
ID	Keywords Plus®	Keywords associated to the manuscript by SCOPUS and Thomson Reuters’ ISI Web of Knowledge databases. Index terms automatically generated from the titles of cited articles.	Similar to Author Keywords, both complement each other.
CR	Cited References	The Cited References count displays the number of documents cited by the current record.	Bibliographic analysis to identify fundamental works, study citation networks, and assess the scientific impact of research.
JI	ISO Source Abbreviation	Scientific journal abbreviations.	Identification and assessment of scientific journals, analysis of publication distribution by journal.
AB	Abstract	Brief summary or description of the essential content from the source document.	Content analysis, keyword extraction, identification of research trends and main themes.
DI	Digital Object Identifier (DOI)	Digital identifier assigned to a document.	Reference management and data integration, traceability of publications for impact and citation analysis.
SO	Publication Name	Scientific journal name.	Offers a full reading of the journal's name, which may not always be clear from the abbreviations.
TC	Web of Science Core Collection Times Cited Count	Number of articles in the database that cite the current article.	Measurement of article impact, analysis of research influence, and detection of highly cited articles.
DB	Source database	Indicates whether the article comes from the Web of Science database (“ISI”) or Scopus (“SCOPUS”).	Comparison of contributions and visibility between different databases.
TI	Document Title	The full title of the journal article.	Identification of articles, analysis of title trends over time.
PY	Year Published	The year of publication of the paper in a journal.	Temporal and evolutionary analysis of research, identification of periods of growth or change within a field.
SR	Short Reference	Title of the article along with its year of publication and journal. Short tag of the document.	Allows for direct referencing of the specific article, without the need to link between various variables.

The integral elements of a scientific article are interconnected. These interconnections create bipartite networks, which can be depicted as rectangular matrices linking manuscripts to variables, a fundamental structure for network, occurrence, or co-occurrence analysis. Moreover, scientific articles often cite other studies, thus forming citation or coupling networks. Investigating these metrics unveils significant aspects of the respective research system.

The variables mentioned are fundamental for a standard bibliometric analysis, covering a wide range of important information and are chosen specifically to allow for the most comprehensive study possible. Nevertheless, the inclusion of variables such as affiliations, which facilitate the mapping of institutional and geographic collaborations and the examination of research networks would have enriched the analysis. The selection of these variables was predicated on the availability of data from Scopus and Web of Science databases. Variables were omitted if the incidence of relevant information in the articles was too sparse to support a substantive analysis.

**Fig. 1:**
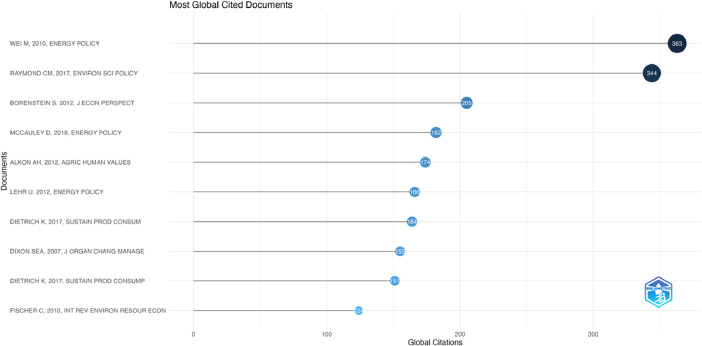
Most Global Cited Documents. This metric reflects how often a document is cited by others within the comprehensive database, such as Web of Science or Scopus. This information is provided by these databases and incorporated into metadata records. This measure serves as an indicator of a document's overall impact across the entire bibliographic database.

**Fig. 2:**
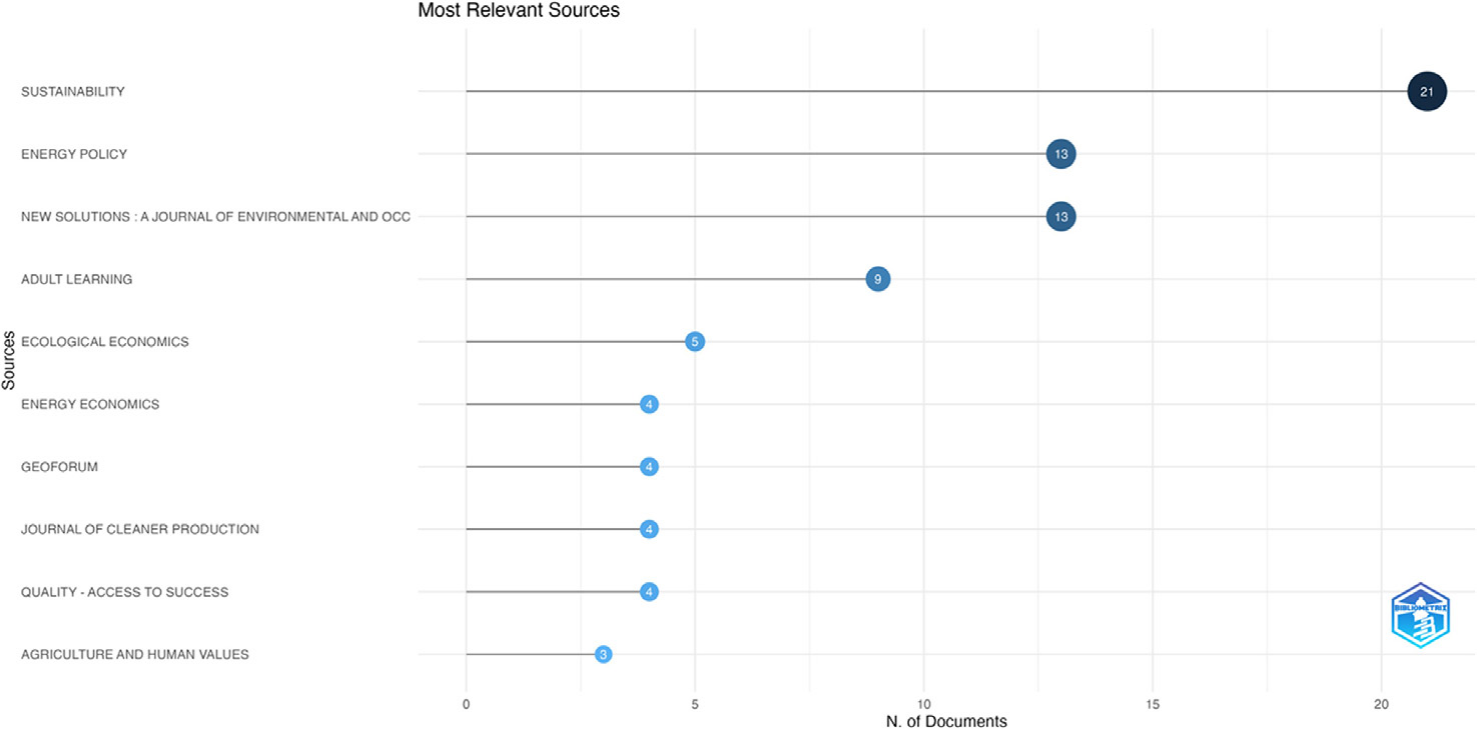
Ten of the most relevant sources. The source is a journal which published one or more documents included in our collection of 268 sources.

**Fig. 3:**
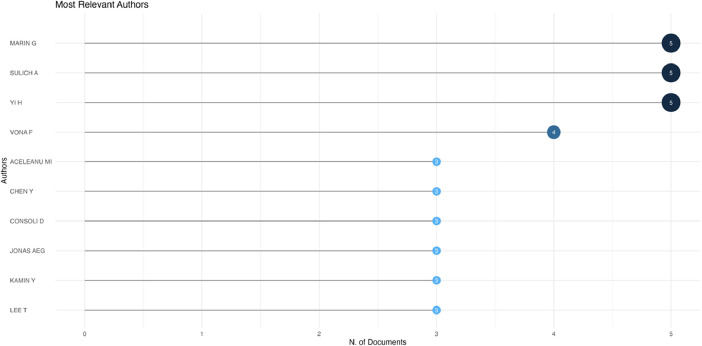
Ten of the most relevant authors. The selected frequency measure is the number of documents per author.

**Fig. 4:**
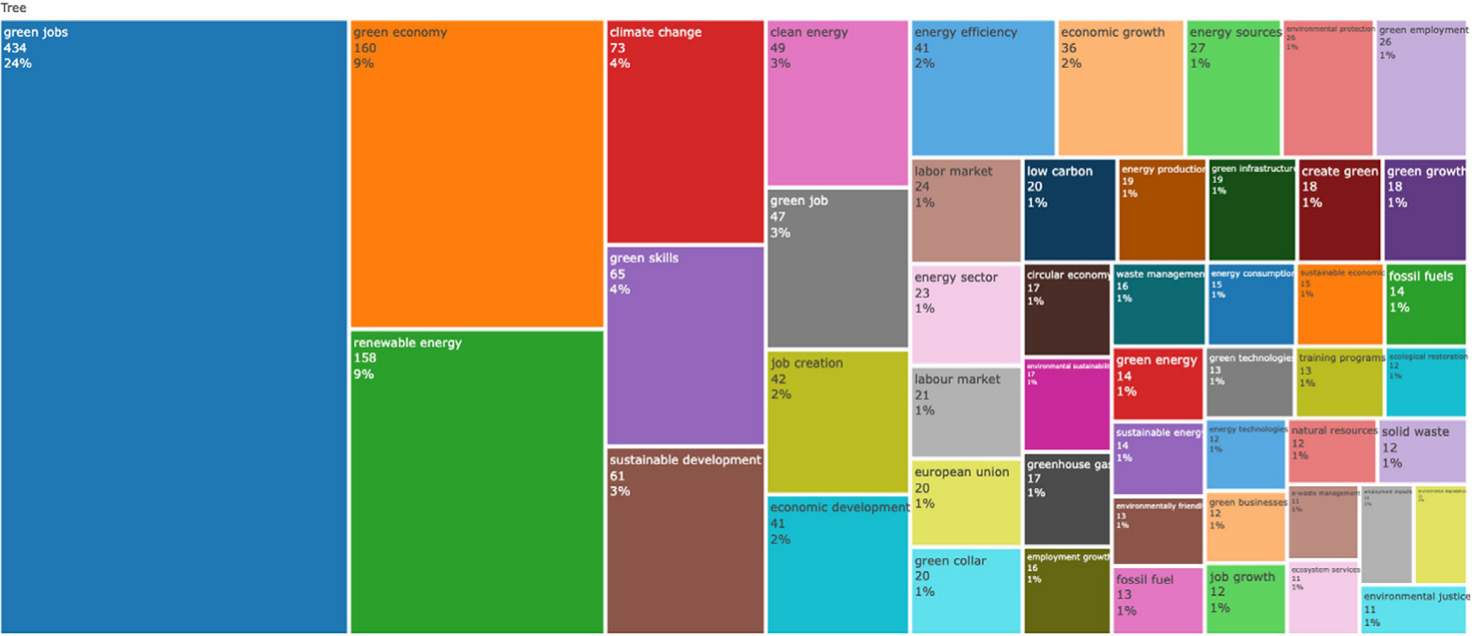
Top title words in papers abstracts represented by Treemap. The N-Grams selected are bigrams.

**Fig. 5:**
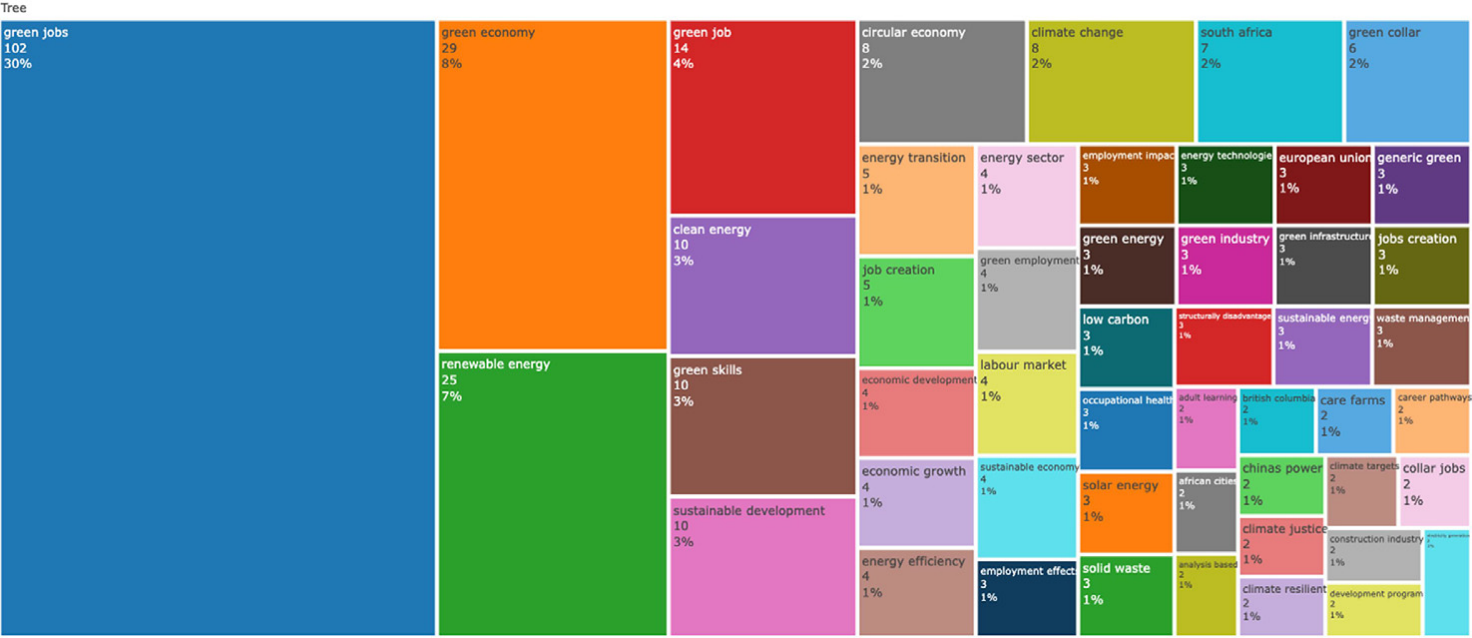
Top title words in papers titles represented by Treemap. The N-Grams selected are bigrams.

**Fig. 6:**
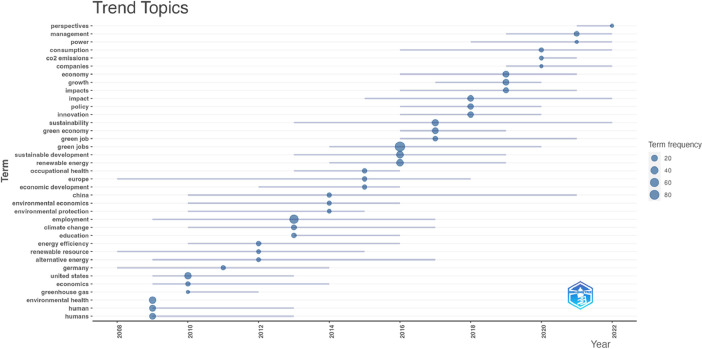
Trend topics derived from Keywords Plus are shown as bubbles, with size indicating occurrence frequency. The grey bar shows the median occurrence distribution. The graph plots time on the horizontal axis and topics on the vertical. The median year of occurrences sets the reference year for each topic. For clarity, only a limited number of the most frequent topics are displayed for each year.

Fig. 1, Fig. 2, Fig. 3, Fig. 4, Fig. 5, Fig. 6 depict bibliometric analyses showcasing the most cited publications, journals, and authors, along with the most frequently occurring terms in abstracts and titles. These summaries provide a preliminary overview of the insights gleaned from this data.

The thorough analysis of the variables contained in this database has led to significant conclusions. For instance, it was possible to identify three major thematic axes guiding the discussion on green jobs: 1) Green jobs in the economic context; 2) The interactions between employment and institutional frameworks; 3) Individual dimensions and social capital. These axes are summarized in Table 2. Furthermore, our analysis has uncovered five key challenges to promote the development of green jobs across a broad range of countries, with particular focus on developing nations. These challenges, summarized in Table 3, include the integration of the informal sector into development policies, the valorization of diverse economic sectors, the development of participatory strategies, the establishment of a well-defined economic framework, and the enhancement of investments in human capital.

**Table 2:** Three thematic groups related to green jobs.

Cluster 1: Green jobs and Economy	Cluster 2: Employment and Institutions	Cluster 3: Human and Social Capital
Themes centered on green economy, renewable energies, technologies, and policies.	Themes at the confluence of policy and education, emphasizing the role of institutions.	Focus on the individual, particularly the worker, with respect to employment dignity and working conditions in green sectors.

**Table 3:** Five challenges for developing green jobs.

Informal Sector	Variety of Sectors	Investment in Human Capital	Bottom-up Approach	Coherent Economic Framework
Essential for catalyzing a greener economy, but also poses risks due to cost-cutting measures that harm the environment. Requires policies to maximize positive aspects and mitigate negative ones.	Involvement in a range of sectors including municipal, energy, agriculture, and more is crucial. Each sector's potential depends on the country's context and requires tailored approaches.	Crucial for promoting green jobs, particularly in developing women's work and gender equality. Requires policies, technologies, training, and financing.	Necessary to consider small-scale technologies and daily ecological practices, especially in the informal sectors of African cities.	Must be backed by robust action ideologies and involve citizen participation, emphasizing job quality and environmental sustainability.

## 4 Experimental Design, Materials and Methods

During the data collection process, the “Preferred Reporting Items for Systematic Reviews and Meta-Analysis” (PRISMA) protocol was adhered to, ensuring a meticulous selection of articles. This methodology is widely endorsed for enhancing the transparency, clarity, and quality of literature reviews [3,4]. The PRISMA protocol unfolds over four distinct stages, in line with the guidelines set by Moher et al. (2009) [4].

1. Identification

This approach merges the Scopus and Web of Science databases, distinguishing itself from many literature reviews that rely on a single source. The method of integrating multiple databases is scarcely found in existing literature [5]. Several works have highlighted the unique strengths of each database, such as the extensive temporal coverage of Web of Science [6] or the broad range of publications in Scopus [6,7]. Although Scopus and Web of Science show a strong correlation [8], many researchers have emphasized the value of analyzing both concurrently, as their data optimally complement each other [5,9,10]. Integrating the two datasets can pose challenges, especially due to variations in article information depending on whether the source is Scopus or Web of Science [5]. A method to merge the Scopus and Web of Science databases has been replicated using the R 'Bibliometrix' package and Excel [11,12]. With this in mind, initial searches on Scopus and Web of Science were conducted separately before merging the data during the second phase of the PRISMA protocol.

Articles mentioning the terms 'Green jobs' or similar expressions in their title, abstract, or keywords were sought (see Table 2). Using site-specific search strings, both singular and plural forms of the expressions were considered (Table 4).

2. Screening

**Table 4:** Bibliographic databases and Keywords.

Bibliographic database	Search data	Search string	Results
Web of Science	10/18/2022	TS=(“Green Job*” OR “Green collar job*” OR “Green employment*” OR “Green work”)	*N* = 507
Scopus	10/18/2022	TITLE-ABS-KEY(“Green Job*” OR “Green collar job*” OR “Green employment*” OR “Green work*”)	*N =* 818

The literature under review is exclusively in English and isn't confined to any specific time frame. Only articles from scientific journals were considered, whether they were research papers, communications, or technical notes. Following an initial screening, 865 articles from both databases were identified. A preliminary review of titles and abstracts led to further refinement. At this stage, the aim was to address the question: “Does this article tackle the issue of green jobs as a distinct employment category addressing the challenges of sustainable transition?”

The search terms used in our queries could sometimes introduce biases. For instance, some of the selected articles referred to the “green jobfish”, a specific fish species. Other articles touched upon “green workplaces,” focusing not on the nature of the job but on environmental aspects of the workspace, such as greenery. Furthermore, all articles centered on “Green Human Resource Management (HRM)” were set aside, as they delve more into creating an eco-friendly work environment rather than the essence of the job itself. After this refinement, 208 articles were discarded. By merging results from Scopus and Web of Science and removing duplicates, we arrived at a consolidated dataset of 440 articles.

3. Eligibility

After establishing our consolidated dataset and creating the corresponding Excel file, we removed articles that had insufficient information or were inaccessible to us. Articles deemed outside our scope upon review were also set aside. At this juncture, 26 articles were excluded from our analysis.

4. Inclusion

**Fig. 7:**
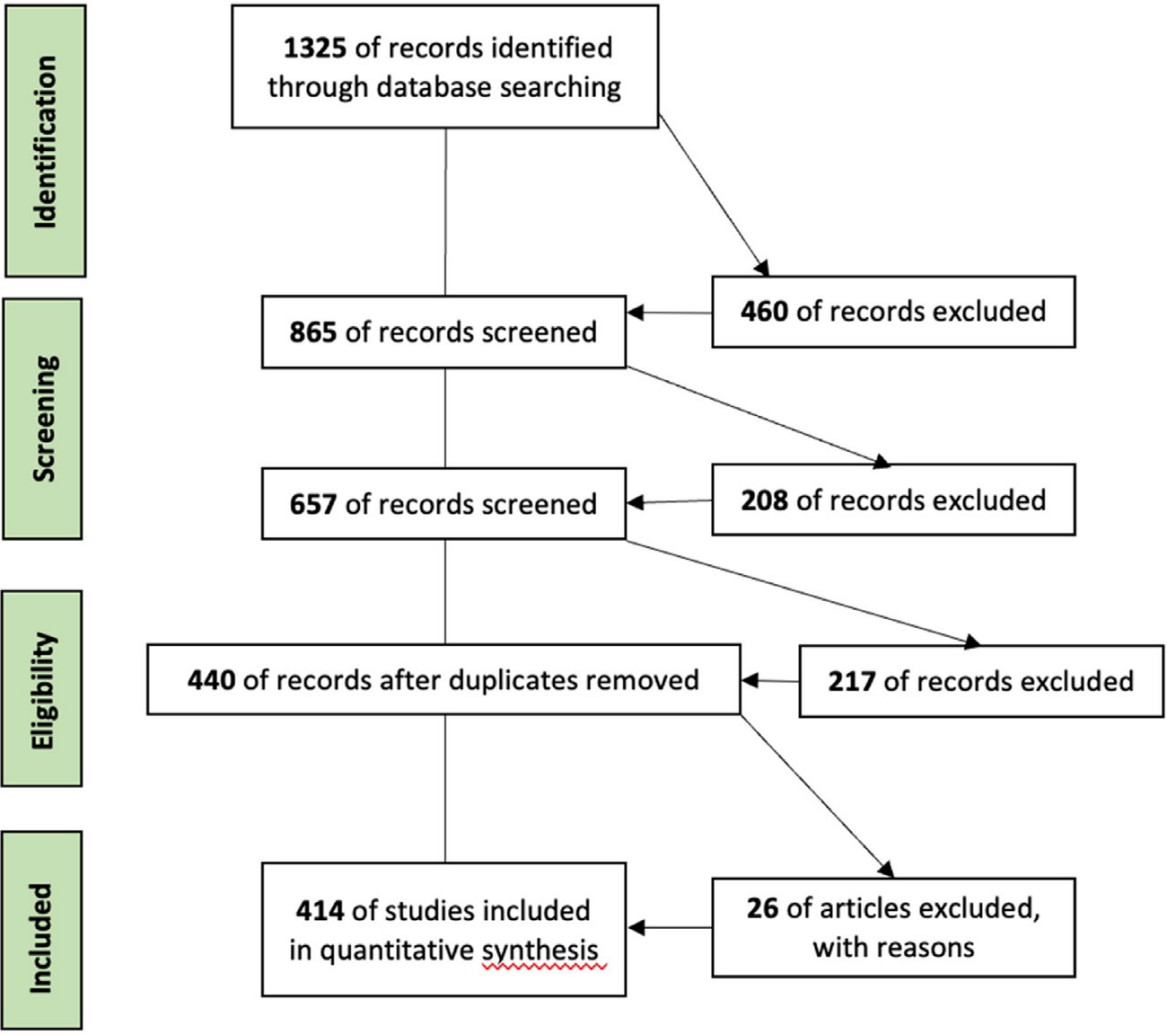
Flowchart of PRISMA procedures and results at each stage.

We thus have a dataset comprising 414 articles. It's worth noting that some articles lack keywords, while others are without an abstract. However, we chose to retain them in our bibliometric analysis, as the other provided information appeared relevant to us (Fig. 7).

Table 5, sourced directly from the Biblioshiny interface of the R package Bibliometrix, depicts the completeness of the metadata, highlighting the missing cells, their percentage, and an assessment of completeness categorized as “Excellent”, “Good”, “Acceptable”, “Poor”, and “Completely missing”. Variables labeled as “Completely missing” were either not initially selected or were not available in the Scopus and Web of Science databases.

**Table 5:** Completeness of the BDGJ metadata.

Metadata	Description	Missing Counts	Missing %	Status
AU	Author	0	0.0%	Excellent
SO	Journal	0	0.0%	Excellent
PY	Publication Year	0	0.0%	Excellent
TI	Title	0	0.0%	Excellent
TC	Total Citation	0	0.0%	Excellent
AB	Abstract	14	3.38%	Good
ID	Keywords Plus	39	9.42%	Acceptable
CR	Cited References	40	11.84%	Acceptable
DI	DOI	70	16.91%	Acceptable
DE	Keywords	92	22.22%	Poor
C1	Affiliation	414	100.00%	Completely missing
RP	Corresponding Author	414	100.00%	Completely missing
DT	Document Type	414	100.00%	Completely missing
LA	Language	414	100.00%	Completely missing
NR	Number of Cited References	414	100.00%	Completely missing
WC	Science Categories	414	100.00%	Completely missing

## Limitations

The data in this document have several limitations. Firstly, they are based on a set of references that is not comprehensive. The exclusive reliance on English literature, the strict selection of articles, and the constraint of the search to a predefined list of keywords might limit the scope of the dataset. Moreover, while Scopus and Web of Science offer a vast collection of well-referenced articles, their access to non-English journals is limited. Additionally, it would be crucial to delve into the grey literature to understand the developmental strategies of this concept at institutional, political, and media levels. These constraints suggest the need to broaden the research, especially by considering other languages and literature sources.

## Ethics Statement

This work complies with the ethical requirements for publication in Data in Brief. This data does not incorporate studies involving animals or humans, nor data gathered from individual social media accounts. The primary data sources used in our study needed no specific permissions and comply with relevant ethical and legal standards.

## CRediT authorship contribution statement

**Alexandre Mathieu:** Conceptualization, Methodology, Investigation, Software, Data curation, Writing – original draft, Validation, Writing – review & editing.

## Data Availability

BDGJ - Bibliometric Dataset on Green Jobs (Original data) (Recherche Data Gouv).
